# The m6A Methyltransferase METTL14-Mediated N6-Methyladenosine Modification of PTEN mRNA Inhibits Tumor Growth and Metastasis in Stomach Adenocarcinoma

**DOI:** 10.3389/fonc.2021.699749

**Published:** 2021-08-12

**Authors:** Qi Yao, Lanzhen He, Xucan Gao, Na Tang, Lifen Lin, Xiaofang Yu, Dong Wang

**Affiliations:** ^1^Department of Anorectal Surgery, The Second Clinical Medical College, Jinan University (Shenzhen People’s Hospital), Shenzhen, China; ^2^Department of Anorectal Surgery, The First Affiliated Hospital, Southern University of Science and Technology, Shenzhen, China; ^3^Department of Colorectal Surgery, The Sixth Affiliated Hospital, Sun Yat-sen University, Guangzhou, China; ^4^Guangdong Provincial Key Laboratory of Colorectal and Pelvic Floor Diseases, The Sixth Affiliated Hospital, Sun Yat-sen University, Guangzhou, China; ^5^Department of Pathology, The Second Clinical Medical College, Jinan University (Shenzhen People’s Hospital), Shenzhen, China; ^6^Department of Pathology, The First Affiliated Hospital, Southern University of Science and Technology, Shenzhen, China; ^7^Department of General Surgery, The Second Clinical Medical College, Jinan University (Shenzhen People’s Hospital), Shenzhen, China; ^8^Department of General Surgery, The First Affiliated Hospital, Southern University of Science and Technology, Shenzhen, China

**Keywords:** METTL14, STAD, Pten, m6A modification, tumor growth, metastasis

## Abstract

**Background:**

Stomach adenocarcinoma (STAD) is a common reason for tumor-related fatalities globally, as it results in distant metastasis. Methyltransferase-like 14 (METTL14), a notable RNA N6-adenosine methyltransferase (m6A), plays a significant role in the growth of tumor through controlling the RNA working. This study aims to highlight METTL14 in STAD’s biological function and molecular mechanism.

**Methods:**

Bioinformatics and immunohistochemical (IHC) assays have been utilized for the detection of METTL14 expression in the STAD. METTL14’s biological function has been shown while making use of HGC-27 and AGS cells *in vitro* experiments. MeRIP-qPCR and luciferase reporter assays were employed for the exploration of METTL14’s mechanism modifying the target of phosphatase and tensin homologue (PTEN). Subcutaneous xeno transplantation model and STAD liver metastasis orthotopic tumor model were used to study METTL14 in STAD *in vivo*.

**Results:**

METTL14 expression was substantially downregulated in STAD reflecting contribution to major tumors, progressed TNM stage as well as poor overall survival (OS) in STAD. Moreover, METTL14’s inhibition of STAD cells proliferation, migration and invasion has been verified *in vitro* assays. Furthermore, an identification of PTEN being METTL14-mediated m6A modification’s substrate has been made. METTL14’s overexpression highly enhanced PTEN mRNA m6A variation, stabilized PTEN mRNA and increased protein expression. Further, it has been found out that METTL14-mediated STAD cells inhibition of proliferation and invasion dependent on PTEN. At last, we demonstrated that METTL14 inhibit STAD growth and metastasis *in vivo* models.

**Conclusions:**

METTL14 inhibits tumor growth and metastasis of STAD *via* stabilization of PTEN mRNA expression. Therefore, METTL14 is a potential biomarker of prognosis and therapeutic targets for STAD.

## Introduction

Gastric cancer is a fifth highest occurring malignant tumor with mortality rate ranked third globally. In Asia, the mortality rate of gastric cancer is second only to lung cancer (1). According to statistics from the United States, the number of estimated new gastric cancer cases had exceeded 27,600 and caused more than 11,010 deaths in 2020 (2). Stomach adenocarcinoma (STAD) being gastric cancer results from gastric gland cells’s malevolent transformation due to which it has been named adenocarcinoma. Gastric adenocarcinoma has an overall 95% representation of gastric cancer cases (3). Surgery combined with radiotherapy and chemotherapy is currently the main method to deal with gastric cancer, but due to hidden early symptoms, most of it is in the middle and late stages at the time of diagnosis, and there are lower than 20% chances of survival after 5 years (4). Universal resistance to chemotherapy drugs is also one of the main reasons for poor efficacy (4). Precision medicine is a new model of tumor diagnosis and treatment, which has been widely used in various fields of medicine (5). At present, the application of gene-based targeted therapy is gradually increasing, and carcinogenic and tumor suppressor genes contribute to gastric cancer’s targeted therapy.

M6A methylation is widely present in RNA, participates in a variety of cell life activities, and has also been reported being a prominent factor behind the emergence of tumors (6). There are three methylases in the process of m6A methylation: METTL3, METTL14 and WTAP (7). METTL14 (methyltransferase like 14) is an important m6A methylase performing a central part in the process of m6A methylation of RNA (8). It has been considered integral to the emergence or development of a variety of tumors. Previous studies had reported that knocking down METTL3 and METL14 can lead to a reduction in m6A levels, thereby inhibiting the self-renewal of mouse embryonic stem cells (9). Moreover, METTL14 inhibits the potential metastatic ability of hepatocellular carcinoma by regulating the microRNA process that depends on m6A methylation (10). The expression of METTL14 is reduced in lung cancer tissues, and its function in lung cancer has not been clearly studied (11). METTL14 also had been reported as a suppressor for tumor metastasis in colorectal cancer, hinting towards METTL14 being valuable therapeutic target for colorectal cancer (12). In gastric cancer, RNA m6A methylation activated oncogenic Wnt/PI3K-Akt signaling and stimulated malicious GC cells’ phenotypes. Moreover, the bioinformatics analysis result between METTL14 and GC had shown that METTL14 has a central part in GC’s biological process. Nevertheless, the purpose and molecular mechanisms of METTL14 in STAD’s tumor progression is still unclear.

This research has first explored METTL14’s expression and clinical significance in STAD tissues of patient. Moreover, we also studied the biological role of METTL14 *in vitro* and *in vivo*, and identified the critical target and modification site of METTL14 in the growth of STAD. Our study provides key evidences regarding METTL14 being STAD’s an important prognostic and healing target.

## Materials and Methods

### Patients and Samples

The Department of Pathology in Shenzhen People’s Hospital (Shenzhen, China) provided the Human STAD and contiguous nonmalignant tissues. All patients had not been given radiotherapy or chemotherapy prior to surgery. Specimens’ rest part was fixed in the formalin and embedded in paraffin for pathological and immunohistochemical (IHC) analysis. Here, the Shenzhen People’s Hospital Ethics Committee allowed our protocol for research. We acquired informed consent of all the participants.

### IHC Analysis

Tissue sections were deparaffinized, rehydrated, and microwaved-heated in sodium citrate buffer (10 mmol/L, pH 6.0) for antigen retrieval. Next followed the incubation of slides with primary antibody (Mouse monoclonal [CL4252] to METTL14, 1:100, Abcam, ab220030), PTEN (1:100, #9188, CST). After that, 02 self-regulating pathologists unaware of patients’ clinical characteristics analyzed target proteins in tissue’s expression levels based on cell staining proportion (0 = 0%, 1 = ≤25%, 2 = 26 to 50%, 3 = 51 to 75%, 4 = >75% positive cells) as well as staining intensity (0 = no staining, 1 = weak, 2 = moderate, 3 = strong). Multiplication of both scores gave the overall results. Protein expressions have been found to be high (≥6) and low (≤6) in cases of final score.

### Cell Culture and Transfection

The Chinese Academy of Sciences (Shanghai, China)’s cell bank is the source of three human STAD cell lines, BGC823, HGC27, and AGS, and human gastric mucosal epithelial cell line (RGM-1). For cell culture, RPMI-1640 (Hyclone) containing 10% FBS (Gibco) have been utilized. Further, the incubation of these cells was performed using humidified incubator with 5% CO_2_ at 37°C.

The METTL14 cDNA’s overall length was synthesized using RBGI Genomics company, and then sub-cloned into the pcDNA3.1 vector to construct pcDNA-METTL14 overexpression (METTL14-OE) plasmid. For transient transfection with plasmids encoding target, Lipofectamine 3000 reagent (Invitrogen) was utilized. For METTL14 stable overexpression in STAD cell lines, the METTL14 gene was constructed into lentiviral expression vector; empty vector worked being the control (Shanghai Genechem). Lentiviral infection was performed following manufacturer’s recommendations. A selection of stably transfected cell lines over 2 weeks was made employing puromycin (2 μg/ml). Moreover, western blotting and RT-PCR was employed to validate infected cells’ transfection efficiency.

### Cell Proliferation Assays

For Cell Counting Kit-8 (CCK-8) assay, transfected cells were inoculated at 2 × 10^3^ cells/well density into 96-well plates and cultivated for 0, 24, 48, and 72 h. Following varying incubation times, 10 μl of CCK-8 reagent (Dojindo Laboratories) was added to all with 2 h continuous culturing. At 450 nm, the absorbance was recorded with standard microplate reader. For colony formation assay, 6-well plates were employed for seeding of 300 stable infected cells and 14 days culturing. Then clones were fixed and stained using 0.5% crystal violet followed by counting of colonies.

### Cell Invasion and Migration Assays

Transwell 24-well plates (8-μm pores; Corning, USA) coated with Matrigel (BD) placed in cell culture hood for around 3 h at 37°C were used for Transwell invasion assay. Transwell migration assay used 8-μm pore size polycarbonate membrane chamber only. After transfection, cells resuspended in serum-free medium were plated into the upper chamber while DEME containing 10% FBS was put in bottom. At 8 or 24 h post-incubation at 37°C, lower surface migrated cells were immobilized in methanol, stained by 0.5% crystal violet and a microphone was employed to count them in five random fields.

### Western Blot Analysis

RIPA buffer (Thermo Scientific, 89900) was used to lyse cells for the purpose of isolating overall protein and supplemented with protease inhibitor cocktail (Bestbio, Shanghai, China) and Phenylmethanesulfonyl fluoride (PMSF) (Bestbio, Shanghai, China). The separation of samples was carried out using 10–12% sodium dodecyl sulfate-polyacrylamide gel electrophoresis (SDS-PAGE) and transferred onto a nitrocellulose membrane, which was stalled using 5% skim milk at 4°C overnight and incubated overnight with specific antibodies to METTL14 (1:1,000, ab220030, Abcam), PTEN (1:1,000, #9188, CST); AKT (1:1,000, #4685, CST); Phospho-Akt (Ser473, 1:1,000, #4060, CST). Following, secondary antibodies at 37°C for 1 h were combined with membranes, and imbibed in electro-chemiluminescence solution for imaging. An evaluation of relative protein level was performed after all the earlier mentioned steps had been successfully completed.

### Bioinformatical Analysis

The comparison of mRNA METTL14’s expression levels in STAD and normal gastric tissue was analyzed by Oncomine microarray database (https://www.oncomine.org/resource/main.html#v:18) (13). METTL14 RNA expression level and prognostic value in STAD were collected from The Human Protein Atlas base on The Cancer Genome Atlas (TCGA) dataset (https://www.proteinatlas.org/). Furthermore, the correlation between METTL14 and PTEN expression in STAD were analyzed by GEPIA (http://gepia.cancer-pku.cn/) (14). The potential substrate between METTL14 and phosphatase and tensin homologue (PTEN) were predicted by m6A2Target (http://m6a2target.canceromics.org/#/validation) (15). Potential regulations that binding to PTEN mRNA m6A modification site were analyze by m6A-Atlas tool (http://180.208.58.66/m6A-Atlas/index.html) (16).

### Real-Time Quantitative PCR (RT-qPCR)

TRIzol reagent (Invitrogen, Carlsbad, CA, USA) was employed obeying supplier’s instructions to separate Overall RNA from cells and tissues. Then 2 μg of RNA were reverse-transcribed into complementary DNA (cDNA) using the M-MLV Reverse Transcriptase (Invitrogen). RT-qPCR was conducted on the 7500 Fast RT-qPCR System using SYBR^®^ Premix Ex Taq™ II (Takara, Dalian, China). Main primers patterns were showed in **Supplementary Table 1**. GAPDH served the function of the internal control. The relative gene expression was quantified by 2^−ΔΔCt^ method.

### Dual-Luciferase Reporter Assay

The Dual-Luciferase^®^ Reporter Assay System (Promega) was employing following manufacturer’s guide. METTL14 and PTEN interaction evaluation was conducted through generating wt-PTEN 3′-UTR-Luc and mut-PTEN 3’-UTR-Luc reporter vectors. HGC-27 and AGS Cells underwent co-transfection with METTL14 overexpression vector, wt-PTEN, mut-PTEN, or negative control plasmids using Lipofectamine 3000 reagent (Invitrogen). Moreover, this research carried out the evaluation of luciferase and renilla activities at 48 h post-transfection along with Dual-Luciferase Reporter Assay System. Renilla activity was used to normalize luciferase active state.

### Quantitative Analysis of m6A RNA Methylation of Total RNA

TRIzol (Invitrogen, CA, USA) was used for the purpose of extracting the overall RNA and the details have been provided below, while its quality was determined using NanoDrop (Thermo Fisher Scientific, Waltham, MA, USA). Its m6A modification level was determined employing EpiQuik m6A RNA Methylation Quantification Kit (p-9005; Epigentek Group Inc., Farmingdale, NY, USA) following guidelines. Briefly, assay wells’ were coated with 200 ng RNA together with m6A standard, which was succeeded by capturing and detecting antibody solution. Moreover, the colorimetric quantification of m6A levels was conducted through considering each well’s absorbance at 450 nm (OD450) wavelength. It was followed by carrying out calculations considering standard curve.

### MeRIP-qPCR

Centrifugation column (MiniBEST Universal RNA Extraction Kit; Takara) was utilized for the purpose of obtaining the Intact total RNA and mRNA was then processed using polyATtract mRNA Isolation Systems (Promega Corp.) for its purification. Following, m6A RNA immunoprecipitation (MeRIP) was conducted employing Magna MeRIP m6A kit (17–10, 499, Millipore) following the guidelines of manufacturer. METTL14 (ab252562) or IgG antibodies were further used for RIP-PCR to detect the METTL14 binding PTEN mRNA. Moreover, RT-qPCR’s IP production was then carried out through the procedure highlighted above. RT-qPCR’s primers have been given in **Supplementary Table 1**.

### Animal Models

Animal care, euthanasia, and usage were approved by the Institutional Animal Care and Use Committee of Jinan University (Guangzhou, China). For the purpose of enhancing the overall randomization of the experiment, a random comparison table had been employed. Accordingly, 5-wk-old male nude athymic BALB/c nu/nu mice (Slack, Shanghai, China) were randomly divided into two parts including a control group (NC) and the experimental group METTL14-OE. For developing subcutaneous xeno transplantation model, 5 × 10^6^ HGC-27 cells stably transfected with NC or METTL14 overexpression were subcutaneously incorporated for 5-week-old BALB/c nude mice. The mice experienced euthanasia after 27 days of inoculation and obtained xenografts’s mass was obtained. Tumor volume over three days was obtained. To create mouse STAD liver metastasis orthotopic tumor model, 1 × 10^6^ HGC-27 cells under stable transfection with NC or METTL14 overexpression were added to subserosal gastric wall of BALB/c nude mice. During the post-injection, after the passing of six weeks, a comparison of metastatic nodules in liver of NC and METTL14-OE groups of mice was performed by evaluated in the under the HE staining. Each hepatic lobule of mice was embedding and performed HE staining, and the number of metastatic nodules in liver were evaluated and accounted *via* each hepatic lobule under microscope.

### Statistical Analysis

The statistical-based requirements were fulfilled through the application of GraphPad Prism 7.0 software (GraphPad, Inc. Here, ± standard deviation (SD) denotes experimental data while conducting experiments in triplicate. Student’s *t* test or ANOVA was used for group comparisons evaluation. Spearman’s rank correlation analysis to assess variables’ relations was carried out. Moreover, Kaplan–Meier approach and log‐rank experiment to evaluate survival curves have been conducted. *P <*0.05 refers to statistics-related significance.

## Results

### Downregulated METTL14 Correlated With Poor Prognosis in STAD

**Figure 1:**
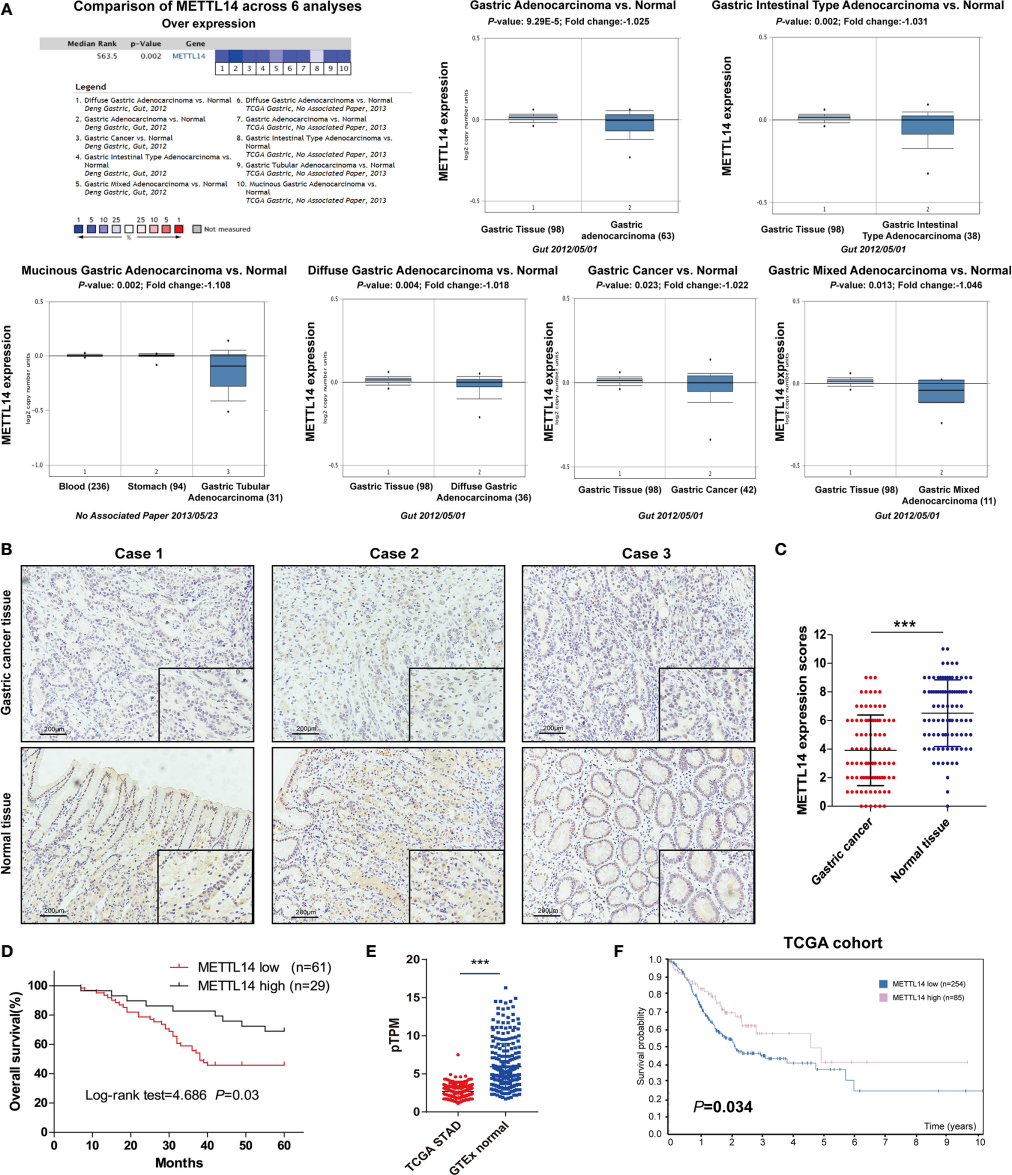
Downregulated METTL14 correlated with poor prognosis in STAD. **(A)** Expression level of METTL14 were analyzed by Oncomine database; **(B)** Representative pictures of immunohistochemical results of METTL14 in STAD and normal tissue. **(C)** Comparision of METTL14 in STAD tissue with normal gastric tissue (n = 90) (***P < 0.001). **(D)** Overall survival comparison between METTL14 high and low expression in STAD patient. **(E)** Comparison METTL14 mRNA expression levels between TCGA STAD and GTEx stomach normal tissue (***P < 0.001). **(F)** Prognostic value of METTL14 mRNA expression in STAD of TCGA dataset.

To investigate m6A’s potential role in STAD, we first discovered that under expressed of METTL14 mRNA expression in STAD tissue takes placed in comparison to normal tissue (**Figure 1A**). Oncomine database result verification was conducted employing the IHC to evaluate the METTL14 expression in the STAD tissue and matched adjacent normal tissue base on 90 clinical specimens, and METTL14 were found that mainly express in nucleus of the STAD cells (**Figure 1B**). Moreover, METTL14 was substantially less expression in STAD tissue compared to usual gastric tissue (**Figure 1C**). Interesting, as shown in **Table 1**, STAD tissue with low METTL14 expression had a significant higher proportion with the larger tumor size (≥5 cm) and III/IV TNM stage. But there is no significant correlation between METTL14 expression and age, gender, differentiation grade and the status of MSI/MMR in STAD patients (**Table 1**). Low METTL14 expression had a correlated with poor levels of the survival (OS) (**Figure 1D**). The RNA expression and prognostic value in STAD from TCGA dataset also were consistent with our clinical data (**Figures 1E, F**). Therefore, our results cleared that METTL14 was downregulated and correlated with poor prognosis in STAD.

**Table 1:** Correlations between METTL14 expression and clinicopathological characteristics in GC.

Parameters	No	METTL14		*P*-value
		High (n = 29)	Low (n = 61)	
Age (years)				0.711
<60	46	14	32	
≥60	44	15	29	
Gender				0.570
Male	38	11	27	
Female	52	18	34	
Differentiation grade				0.153
Well/moderate	59	16	43	
Poor/undifferentiated	31	13	18	
Tumor size (cm)				0.018
<5	49	21	28	
≥5	41	8	33	
TNM stage				0.026
I/II	60	24	36	
III/IV	30	5	25	
MSI/MMR				0.754
pMMR/MSI-L/MSS	79	25	54	
dMMR/MSI-H	11	4	7	

pMMR, proficient mismatch repair; MSI-L, microsatellite instability low; MSS, microsatellite stable; dMMR, deficient mismatch repair; MSI-H, microsatellite instability high.

### Overexpression of METTL14 Inhibited Proliferation and Invasion of STAD

**Figure 2:**
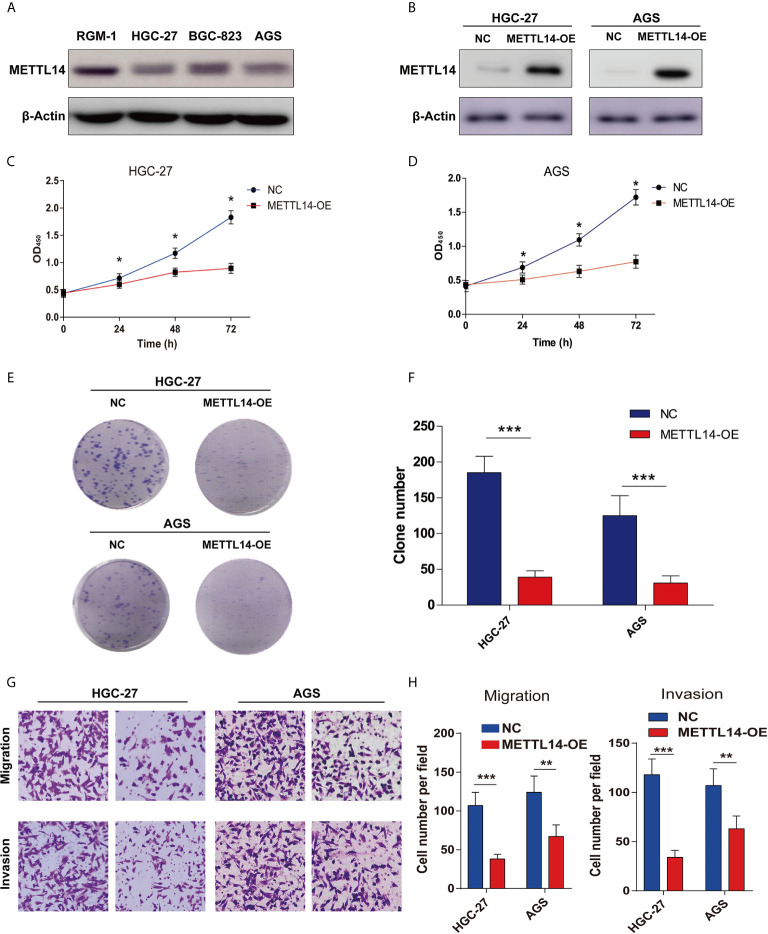
Overexpression of METTL14 inhibited proliferation, migration and invasion of STAD. **(A)** METTL14 was lower expression in STAD cell lines (HGC-27, BGC-823, AGS) compared with normal gastric mucosa cell line RGM-1. **(B)** Validation the expression level of METTL14 after the over expression METTL14 in HGC-27 and AGS cells. **(C**, **D)** CCK8 were used to detect the cell viability between negative control (NC) and METTL14 overexpression (METTL14-OE) in HGC-27 and AGS cells (*P < 0.05). **(E**, **F)** Colony formation assay were used to detect the proliferation of HGC-27 and AGS cells after METTL14 overexpression (***P < 0.001). **(G, H)** Transwell assay were used to detect the migrative and invasive ability of HGC-27 and AGS cells after METTL14 overexpression (**P < 0.01; ***P < 0.001).

Since METTL14 is down-regulated and correlated with tumor growth and TNM stage in STAD, an assumption was made that METTL14 could perform the function of tumor suppressor in STAD. For outlining METTL14 in STAD’s functional role, firstly, an evaluation of the METTL14 expression level in normal gastric mucosa cell line RGM-1, and STAD cell lines HGC-27, BGC-823 and AGS. Obviously, METTL14 had lower expression in STAD cell lines in comparison to those of the normal gastric mucosa cell line RGM-1 (**Figure 2A**). Moreover, we established METTL14 overexpression (METTL14-OE) cell model in HGC-27 and AGS cells (**Figure 2B**). As expected, METTL14’s overexpression markedly stalled cell viability and STAD cells’ proliferative capability as can be realized from the elevated growth rate (**Figures 2C, D**) and colony number rise (**Figures 2E, F**) of METTL14-OE cells. Further, transwell invasion assay highlighted overexpression of METTL14 dramatically inhibiting migrative in STAD cells (**Figures 2G, H**). Resultantly, overexpression of METTL14 inhibited STAD cells proliferation and invasion *in vitro*.

### METTL14 Regulated PTEN Expression in STAD

**Figure 3:**
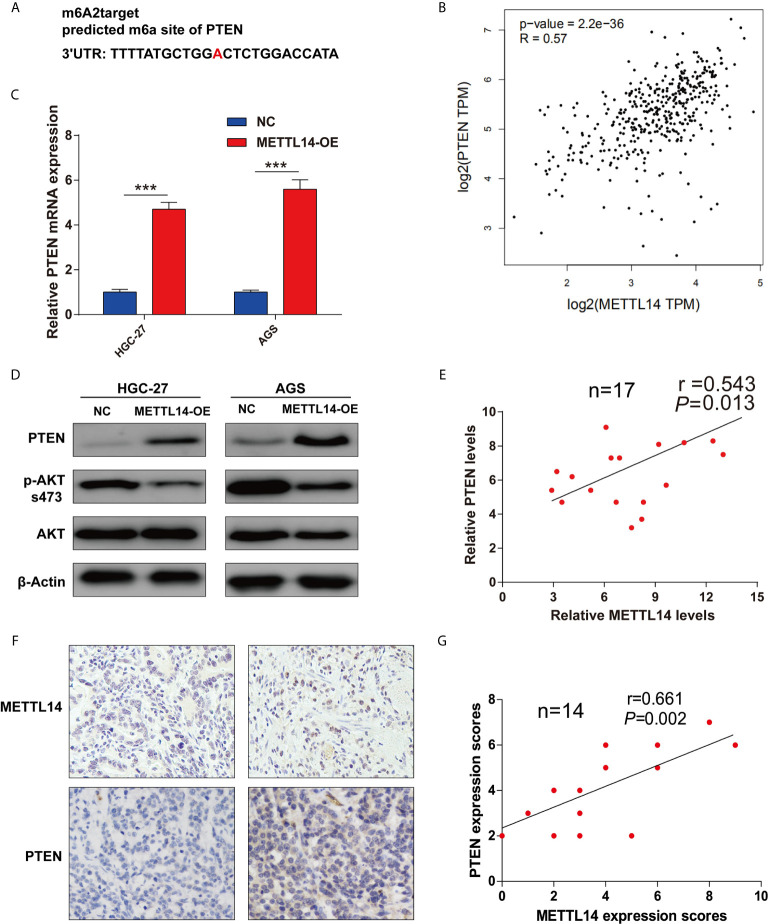
METTL14 regulated PTEN expression in STAD. **(A)** The potential target site of PTEN modified by METTL14 was predicted by m6A2Target. **(B)** Correlation analysis between METTL14 and PTEN expression were performed by GEPIA database. **(C)** PTEN, AKT expression level, and AKT phosphorylation (Ser473) level were detected after METTL14 overexpression in HGC-27 and AGS cells (***P < 0.001). **(D, E)** Correlation analysis between METTL14 and PTEN mRNA expression in STAD tissue (n = 17); **(F**, **G)** Correlation analysis between METTL14 and PTEN protein expression in STAD tissue by IHC (n = 14).

To further study METTL14’s mechanism in proliferation and invasion of STAD, firstly, a prediction of potential modified substrates in m6A2Target database was made. Interesting, PTEN was a verified modified substrate of METTL14 in A549 cell lines (**Figure 3A**). However, the relationship between METTL14 and PTEN in STAD is still unclear. Therefore, we analyzed the correlation of mRNA expression between METTL14 and PTEN in STAD *via* GEPIA database. The result showed that METTL14 expression level is significantly correlated with PTEN expression level in STAD (**Figure 3B**). Overexpression METTL14 enhanced PTEN mRNA expression in HGC-27 and AGS cells (**Figure 3C**). Moreover, we detected the PTEN expression level and AKT’s phosphorylation level, the downstream pathway of PTEN. Our result was clear that METTL14 overexpression enhanced PTEN expression and phosphorylation level of Ser473 of AKT in HGC-27 and AGS STAD cell lines (**Figure 3D**). An analysis of mRNA and protein expression levels between METTL14 and PTEN in STAD tissue was conducted, and METTL14 also positively correlated with PTEN expression in mRNA and protein level in STAD (**Figures 3E–G**). Collectively, these results suggested METTL14 is a critical m6A methylase that regulating PTEN expression in STAD.

### METTL14 Stabilize PTEN mRNA *via* m6A Modification in STAD

**Figure 4:**
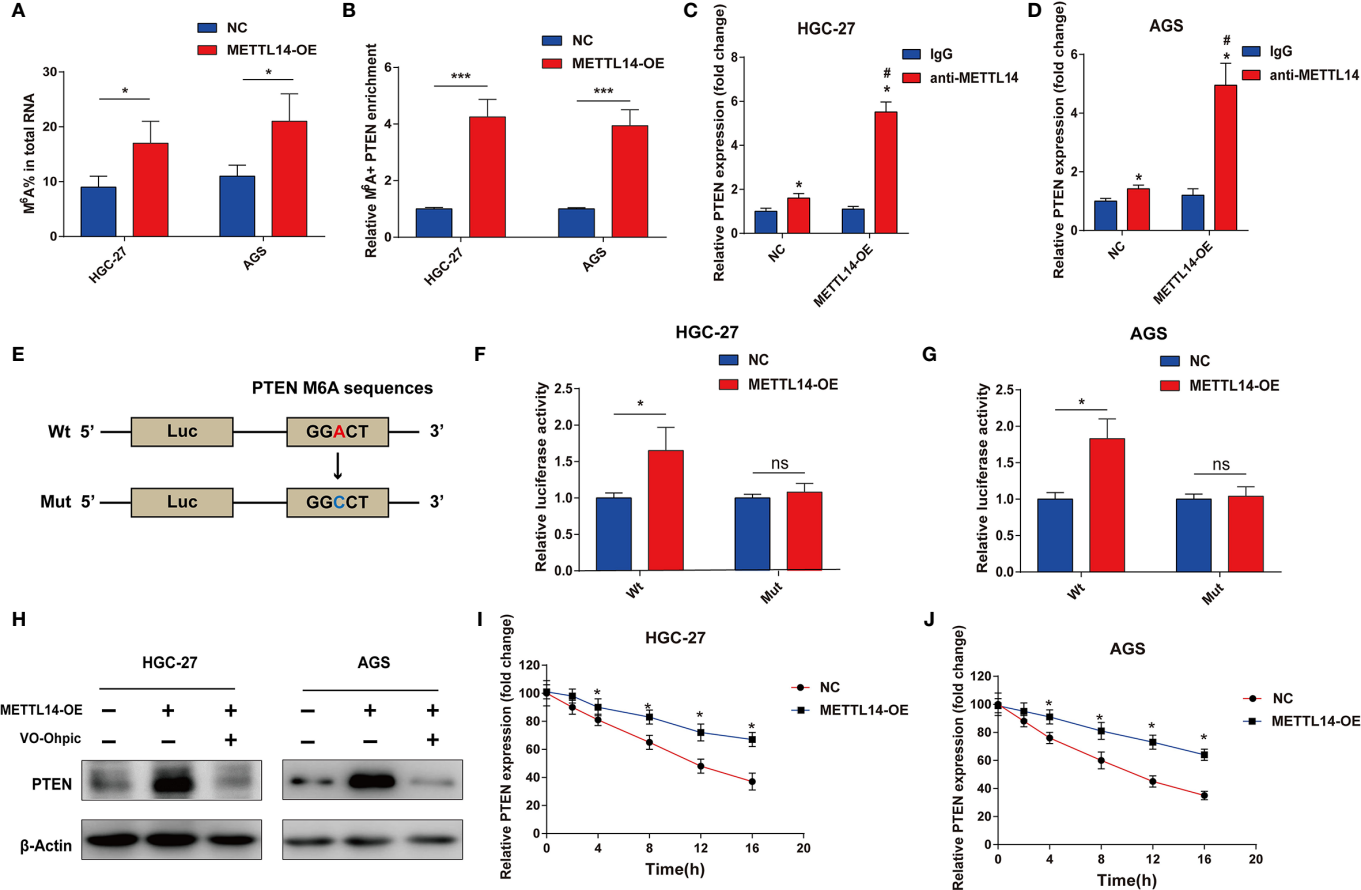
METTL14 regulates PTEN mRNA expression *via* m6A modification in STAD. **(A)** M6A modification level of total RNA was detected after METTL14 overexpression in HGC-27 and AGS cells. **(B)** Enrichment of m6A in PTEN was detected after METTL14 overexpression in HGC-27 and AGS cells (***P < 0.001). **(C**, **D)** RIP-qPCR assay were used to detect the enrichment of METTL14 binding PTEN in HGC-27 and AGS cells (*, *vs* IgG, *P < *0.05; #, *vs* anti-METTL14 in NC group, *P < *0.05). **(E)** Luciferase reporters of wild type and mutation PTEN were constructed. **(F**, **G)** Relative luciferase activity were detected after transfecting wt-PTEN 3′-UTR-Luc or mut-PTEN 3’-UTR-Luc reporter vectors in METTL14 overexpression or negative control STAD cells *P < 0.05; ns, no significant difference. **(H)** METTL14 overexpression mediating PTEN upregulation was antagonized by the PTEN inhibitor, VO-Ohpic, in HGC-27 and AGS cells. **(I**, **J)** The overexpression of METTL14 extended the half-life of PTEN mRNA in HGC-27 and AGS cells. *P < 0.05.

For METTL14 targets PTEN mRNA *via* m6A modification verification, m6A’s global level in control and METTL14 overexpression groups were determined (**Figure 4A**). Accordingly, there was a notable rise of the overall METTL14’s overexpression in HGC-27 and AGS cells. Then, the measurement of MeRIP-qPCR’s enrichment of m6A in PTEN was carried out. We have found out that the m6A abundance in the PTEN mRNA was dramatically increased on METTL14 overexpression (**Figure 4B**). Furtherly, we also confirmed the METTL14 whether binding to PTEN in HGC-27 and AGS cells. Overexpression METTL14 significantly enhances the enrichment of METTL14 binding PTEN mRNA in HGC-27 and AGS cells *via* RIP-qPCR (**Figures 4C, D**). For m6A modification impacts on PTEN expression, luciferase reporters having either wild-type or mutant PTEN was used. In the case of PTEN’s mutant form, cytosine was used instead of adenine bases in m6A consensus sequences (GGACT) resulting in eliminating m6A modification (**Figure 4E**). Luciferase reporter assay showed wild-type PTEN’s transcriptional level though not mutation, clearly decreased in METTL14’s overexpression (**Figures 4F, G**). METTL14 overexpression mediating PTEN upregulation was antagonized by the PTEN inhibitor, VO-Ohpic, in HGC-27 and AGS cells (**Figure 4H**). This has shown PTEN level’s regulation had been within the influence and control of METTL14-related m6A modification. We further supplemented haft-time of mRNA after METTL14-OE in HGC-27 and AGS cells. In addition, we measured the loss of PTEN mRNA in HGC-27 and AGS cells treated with α-amanitin, an inhibitor of RNA synthesis. We found that overexpression of METTL14 extended the half-life of PTEN mRNA in HGC-27 and AGS cells (**Figures 4I, J**). At last, we predicted the binding proteins of m6A modification site of PTEN mRNA using m6A-Atlas tool. Interesting, we found IGF2BP2 and IGF2BP3, the m6A readers that play critical roles for enhancing the stability and storage capacity of mRNA, were binding proteins of m6A modification site on PTEN mRNA (**Figure S1A**). Then we analyzed the correlation between the mRNA expression between PTEN and IGF2BP2 or IGF2BP3. PTEN was positively correlated with IGF2BP2 and IGF2BP3 in STAD (**Figures S1B, C**). Therefore, METTL14 improve mRNA stability of PTEN in STAD.

### METTL14 Inhibits STAD Cells Proliferation and Invasion *via* Regulation of PTEN

**Figure 5:**
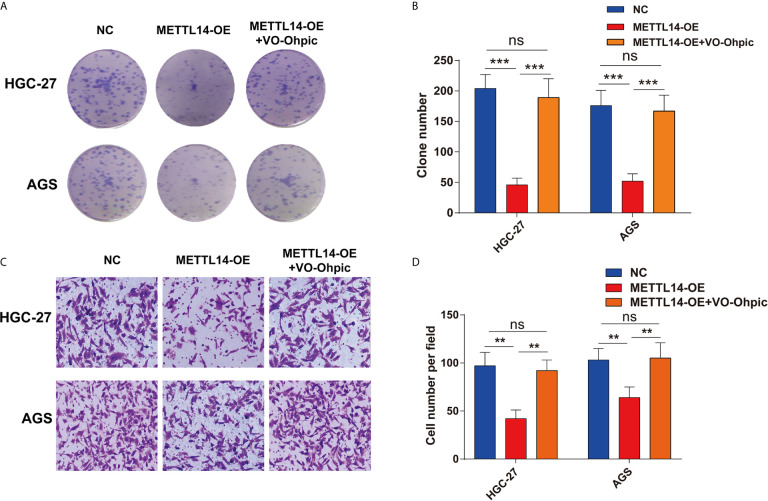
METTL14 inhibits STAD cells proliferation and invasion *via* regulation of PTEN. **(A**, **B)** METTL14 overexpression inhibits HGC-27 and AGS cells growth, and targeting PTEN by VO-Ohpic antagonized the effect of METTL14 inhibiting the growth of HGC-27 and AGS cells. **(C**, **D)** METTL14 overexpression inhibits HGC-27 and AGS cells invasion. When METTL14 overexpression and treatment of VO-Ohpic in HGC-27 and AGS cells, the invasive abilities of HGC-27 and AGS cells after METTL14 overexpression was enhanced by VO-Ohpic. (**P < 0.01; ***P < 0.001; ns, no significant difference).

The current research has revealed modifying effects of METTL14’s depletion on PTEN’s expression in STAD. However, whether METTL14 inhibits STAD cells proliferation and invasion *via* PTEN was still not clear. VO-Ohpic is a highly effective PTEN inhibitor, here, we overexpress METTL14 combined with treatment of VO-Ohpic (2 μM) in HGC-27 and AGS cells. Interesting, VO-Ohpic significantly antagonized the inhibitation effect of METTL14 in the HGC-27 and AGS cells colony formation (**Figures 5A, B**). Moreover, overexpress METTL14 and inhibit PTEN expression for HGC-27 and AGS cells that significantly eliminated METTL14’s inhibitation effect of HGC-27 and AGS cells invasive ability (**Figures 5C, D**). Therefore, it has further been confirmed that METTL14 inhibits STAD cells proliferation and invasion *via* regulation of PTEN.

### METTL14 Inhibits STAD Growth and Metastasis *In Vivo*

**Figure 6:**
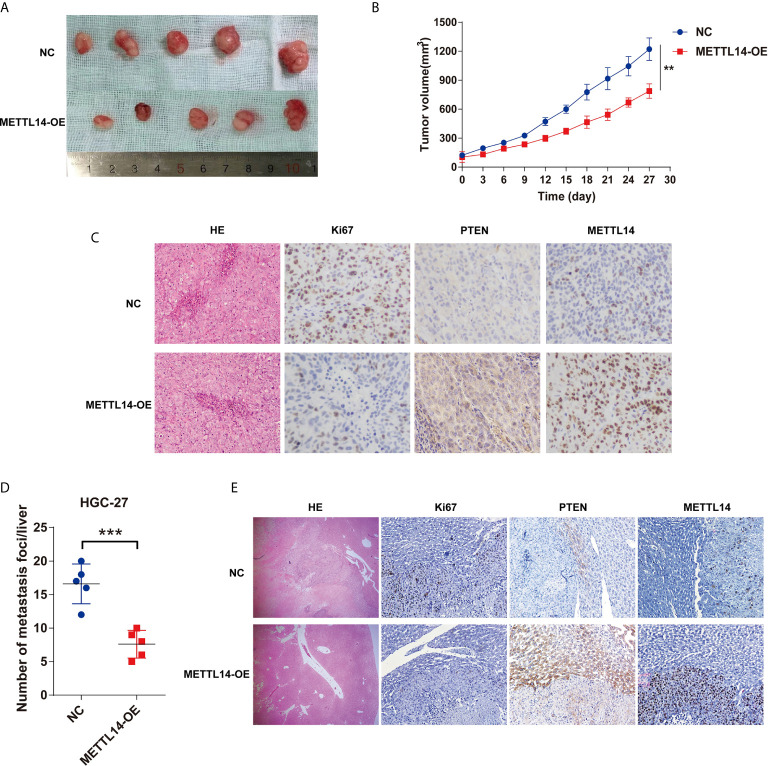
METTL14 inhibits STAD growth and metastasis *in vivo*. **(A)** The representative tumors of METTL14 overexpression or negative control HGC-27 cell after transplanting in nude mice. **(B)** Comparison of tumor volumes of transplanted tumor nude mice between METTL14 overexpression or negative HGC-27 cells. **(C)** Comparison of metastasis foci in liver of STAD liver metastasis orthotopic tumor models between METTL14 overexpression or negative HGC-27 cells. **(D)** The representative pictures of HE-stained, Ki67, PTEN and METTL14 expression in STAD subcutaneous xeno transplantation model. **(E)** The representative pictures of HE-stained, Ki67, PTEN and METTL14 expression in liver metastasis sites of STAD tumor in orthotopic tumor models. (**P < 0.01; ***P < 0.001).

For METTL13 *in vivo* cancer suppressor’s role comprehensive determination, a subcutaneous xenotransplantation model has been utilized for the consideration of METTL13’s effect on STAD growth expression. Tumor volumes were studied for the nude mice that were subcutaneously given METTL14 overexpression HGC-27 cells (METTL14-OE) or control (NC) transfected HGC-27 cells. **Figure 6A** gives tumor volumes at specific time points. Generally, METTL14-OE group’s tumor volume was substantially stalled contrary to that of control group after 27 d post-injection (**Figure 6B**).

A STAD liver metastasis orthotopic tumor model for detecting METTL14’s effect was executed on CRC metastasis *in vivo*. After 6 weeks, metastatic nodules quantity in METTL14-OE group’s livers was lower than control group (**Figure 6C**), the livers and metastatic nodules, and PTEN expression in metastatic nodules (**Figure 6D, E**). Mentioned outcomes further confirmed that METTL14 plays as a cancer suppressor gene in STAD growth and metastasis *in vivo*.

## Discussion

STAD is a gastrointestinal cancer found globally (17). Invasion and metastasis are usual characteristics of progressed STAD and leads to STAD patients’ weak prognosis (18). Despite advanced therapy developments such as surgery, chemotherapy, radiotherapy, and immunotherapy, the patients’ survival rate over five years remains low (19). Therefore, pathological mechanism understanding and particularly proliferation and metastasis for commanding STAD treatment are essential. The highlighted evidence in the research has further shown that M6A methylations are central regulators in human cancers’ pathogenesis such as STAD (20). Previous study reported that the expression of ALKBH5 in highly aggressive diffuse gastric adenocarcinoma was significant higher than that of normal tissue. ALKBH5 stimulates gastric cancer’s invasion and metastasis through lowering lncRNA NEAT1’s methylation (21). These results have highlighted ALKBH5 being a tumor promoting gene in gastric cancer. Moreover, FTO also be found that in the gastric cancer’s progression and metastasis. It has a relationship with weaker differentiation, lymph node metastasis, TNM staging, and poor prognosis, and could be crucial molecular marker for monitoring gastric cancer (22). METTL3 is located in the nucleus and is an enzyme that catalyzes the modification of m6A. METTL3 in the cytoplasm can also function independently of enzyme activity and promote mRNA translation. Although METTL14 is homologous to METTL3, it lacks the catalytic activity domain of the enzyme, which is believed to improve the catalytic activity of METTL3 by providing an RNA binding platform (8). Unlike METTL14, METTL3 had been considered as a cancer-promoting gene in various types of cancer (11). METTL3 has a role in gastric cancer cells’ proliferation and migration regulation, which significantly affects the expression of α-smooth muscle actin, and is expected to become gastric cancer’s target in future (23). However, METTL14’s function in STAD is currently obscure. The current research has further identified METTL14-mediated N6-methyladenosine modification of PTEN mRNA controlling tumor growth and metastasis in STAD, which implied that METTL14 is a potential therapeutic target in STAD.

We firstly identified the lower expression of METTL14 in STAD *via* IHC of clinical specimens. Although previous study showed METTL14 is low expression in gastric cancer, but this result just explored in the database and base on the mRNA level (24). Here, we confirmed the METTL14 was mainly in cell nucleus of the STAD cells, and low METTL14 also correlated to bigger tumor and greater TNM stage, as well as weaker prognosis. These results further confirmed the potential favorable biomarker role of METTL14 in STAD. As previously reported, METTL14 could serve as a suitable prognostic factor for clear renal cell, carcinoma cell, and hepatocellular carcinoma (25, 26). However, downregulated METTL14 expression is just associated with tumor grade and molecular classification, but not associated with patient prognosis in breast cancer (27). Moreover, upregulation of METTL14 promotes the growth and metastasis of pancreatic cancer (28). Therefore, the role of METTL14 in different cancer types has different impacts in the cancer progress.

Secondly, we further explored the role of METTL14 in the biological behavior of STAD cells. We further confirmed that METTL14 as a tumor suppressor in the STAD cells. Overexpression of METTL14 inhibits STAD cells’ propagation, assault, and migration. These results are also consistent with the clinical features of METTL14 in STAD. Moreover, the potential mechanism of METTL14 in suppressing growth and metastasis of STAD was also studied in our research. Based on the predictive substrates of METTL14 in m6A2Target database, PTEN is a potential substrate of METTL14 for m6A modification. Although the previous studies implied that METTL14 plays an important role in the PI3K/AKT signaling pathway (29), further validations and definite mechanisms should be explored in the further studies. Therefore, we verified the relationship between the METTL14 and PTEN in the STAD cells. Interestingly, METTL14 not only positively correlated with PTEN expression in STAD from TCGA database, but also from our verified clinical samples. The western-blot result also confirms the change of downstream proteins after overexpression of METTL14 in STAD cells. At last, we confirmed the m6A modification site of PTEN by METTL14 in STAD cells, and rescue assays also were used to verify the suppressing role of METTL14 in STAD which is dependent on the PTEN. Therefore, we firstly cleared an exact mechanism in the METTL14 suppressing STAD in our study. For more m6A regulation processes of PTEN in STAD, such as the downstream regulator of m6A reader, we also identified that IGF2BP2 and IGF2BP3 were potential binding proteins for the PTEN m6A modification site and positively correlated with PTEN expression. As IGF2BP2 and IGF2BP3 can maintain the stability of mRNAs in m6A modification process (30, 31), our result also indicated METTL14 can extend the half-life of PTEN mRNA in STAD cells. Therefore, IGF2BP2 and IGF2BP3 may be the potential readers for METTL14 regulating the stability of PTEN mRNA.

At last, the *in vivo* results are also consistent with the *in vitro* results of the role of METTL14 in STAD. Our study used the HGC-27 cells to construct the subcutaneous xeno transplantation model and STAD liver metastasis orthotopic tumor model. Overexpression of METTL14 also inhibits STAD growth and liver metastasis *in vivo.* These results also provide important evidences that METTL14 could be a potential target for treatment for STAD. The agonist of METTL14 also has broad clinical application prospects in the treatment of cancer. Moreover, as PTEN is a crucial target in the chemotherapy-resistance and cancer immunotherapy (28), the regulation between METTL14 in the role of chemotherapy-resistance and cancer immunotherapy in STAD should be further studied.

Summarily, the work conducted in this research involving empirical analysis has provided benefits in determining the central role that can be performed by METTL14-mediated m6A modification in human STAD progression and a charming m6A-dependent regulatory mechanism. The results of the current research have shown that METTL14 epigenetically inhibited the expression of PTEN *via* an m6A modification dependent mechanism. This is an important finding that was be exploited for different types of applications in the field and focusing on bringing improvements in the areas that currently lack behind despite substantial development overtime. The METTL14/PTEN axis finding and the overall effect it can create on STAD metastasis will be useful in performing future research in STAD considering the efficient therapeutic techniques, mechanisms, and strategies that can be developed in providing people relief against STAD.

## Data Availability Statement

The raw data supporting the conclusions of this article will be made available by the authors, without undue reservation.

## Ethics Statement

The studies involving human participants were reviewed and approved by Shenzhen People’s Hospital Ethics Committee. The patients/participants provided their written informed consent to participate in this study. The animal study was reviewed and approved by Institutional Animal Care and Use Committee of Jinan University.

## Author Contributions

Conception/design: QY, XY, and DW. Collection and/or assembly of data: LH, NT, LL, and XG. Manuscript writing: QY, XY, and DW. All authors contributed to the article and approved the submitted version.

## Conflict of Interest

The authors declare that the research was conducted in the absence of any commercial or financial relationships that could be construed as a potential conflict of interest.

## Publisher’s Note

All claims expressed in this article are solely those of the authors and do not necessarily represent those of their affiliated organizations, or those of the publisher, the editors and the reviewers. Any product that may be evaluated in this article, or claim that may be made by its manufacturer, is not guaranteed or endorsed by the publisher.

## Abbreviations

STAD, Stomach adenocarcinoma; METTL14, Methyltransferase-like 14; m6A, N6-adenosine methyltransferase; IHC, immunohistochemical; PTEN, phosphatase and tensin homologue; OS, overall survival; CCK-8, Cell Counting Kit-8; PMSF, phenylmethanesulfonyl fluoride.

## References

[1] Serra O Galán M Ginesta MM Calvo M Sala N Salazar R. Comparison and Applicability of Molecular Classifications for Gastric Cancer. Cancer Treat Rev (2019) 77:29–34. 10.1016/j.ctrv.2019.05.005 31195213

[2] Siegel RL Miller KD Jemal A Siegel RL Miller KD. Cancer Statistics. (2020) 2020:7–30. 10.3322/caac.21590

[3] Li J Zhou W Wei J. Prognostic Value and Biological Functions of RNA Binding Proteins in Stomach Adenocarcinoma. Onco Targets Ther (2021) 14:1689–705. 10.2147/OTT.S297973 PMC7942957 33707953

[4] Kurokawa Y Yang HK Cho H Ryu MH Masuzawa T Park SR. Phase II Study of Neoadjuvant Imatinib in Large Gastrointestinal Stromal Tumours of the Stomach. Br J Cancer (2017) 117:25–32. 10.1038/bjc.2017.144 28535156 PMC5520207

[5] Shim JH Yoon JH Choi SS Ashktorab H Smoot DT Song KY. The Effect of Helicobacter Pylori CagA on the HER-2 Copy Number and Expression in Gastric Cancer. Gene (2014) 546:288–96. 10.1016/j.gene.2014.05.064 PMC4286173 24879917

[6] Cantara WA Crain PF Rozenski J McCloskey JA Harris KA Zhang X. The RNA Modification Database, RNAMDB: 2011 Update. Nucleic Acids Res (2011) 39:D195–201. 10.1093/nar/gkq1028 PMC3013656 21071406

[7] Wang X Zhao BS Roundtree IA Lu Z Han D Ma H. N(6)-Methyladenosine Modulates Messenger RNA Translation Efficiency. Cell (2015) 161:1388–99. 10.1016/j.cell.2015.05.014 PMC4825696 26046440

[8] Xu Z Peng B Cai Y Wu G Huang J Gao M. N6-Methyladenosine RNA Modification in Cancer Therapeutic Resistance: Current Status and Perspectives. Biochem Pharmacol (2020) 182:114258. 10.1016/j.bcp.2020.114258 33017575

[9] Wang Y Li Y Toth JI Petroski MD Zhang Z Zhao JC. N6-Methyladenosine Modification Destabilizes Developmental Regulators in Embryonic Stem Cells. Nat Cell Biol (2014) 16:191–8. 10.1038/ncb2902 PMC4640932 24394384

[10] Ma JZ Yang F Zhou CC Liu F Yuan JH Wang F. METTL14 Suppresses the Metastatic Potential of Hepatocellular Carcinoma by Modulating N(6) -Methyladenosine-Dependent Primary MicroRNA Processing. Hepatol (Baltimore Md) (2017) 65:529–43. 10.1002/hep.28885 27774652

[11] Lin S Choe J Du P Triboulet R Gregory RI. The M(6)A Methyltransferase METTL3 Promotes Translation in Human Cancer Cells. Mol Cell (2016) 62:335–45. 10.1016/j.molcel.2016.03.021 PMC4860043 27117702

[12] Chen X Xu M Xu X Zeng K Liu X Pan B. METTL14-Mediated N6-Methyladenosine Modification of SOX4 mRNA Inhibits Tumor Metastasis in Colorectal Cancer. Mol Cancer (2020) 19:106. 10.1186/s12943-020-01220-7 32552762 PMC7298962

[13] Rhodes DR Yu J Shanker K Deshpande N Varambally R Ghosh D. ONCOMINE: A Cancer Microarray Database and Integrated Data-Mining Platform. Neoplasia (New York NY) (2004) 6:1–6. 10.1016/S1476-5586(04)80047-2 PMC1635162 15068665

[14] Tang Z Li C Kang B Gao G Li C Zhang Z. GEPIA: A Web Server for Cancer and Normal Gene Expression Profiling and Interactive Analyses. Nucleic Acids Res (2017) 45:W98–102. 10.1093/nar/gkx247 28407145 PMC5570223

[15] Deng S Zhang H Zhu K Li X Ye Y Li R. M6A2Target: A Comprehensive Database for Targets of m6A Writers, Erasers and Readers. Briefings Bioinf (2020) 22(3):bbaa055. 10.1093/bib/bbaa055 32392583

[16] Tang Y Chen K Song B Ma J Wu X Xu Q. m6A-Atlas: A Comprehensive Knowledgebase for Unraveling the N6-Methyladenosine (m6A) Epitranscriptome. Nucleic Acids Res (2021) 49:D134–43. 10.1093/nar/gkaa692 PMC7779050 32821938

[17] Wu M Xia Y Wang Y Fan F Li X Song J. Development and Validation of an Immune-Related Gene Prognostic Model for Stomach Adenocarcinoma. Biosci Rep (2020) 40(10):BSR20201012. 10.1042/BSR20201012 33112406 PMC7593539

[18] Ye T Yang M Huang D Wang X Xue B Tian N. MicroRNA-7 as a Potential Therapeutic Target for Aberrant NF-κB-Driven Distant Metastasis of Gastric Cancer. J Exp Clin Cancer Research: CR (2019) 38:55. 10.1186/s13046-019-1074-6 30728051 PMC6364399

[19] Zhu L Wang H Jiang C Li W Zhai S Cai X. Clinically Applicable 53-Gene Prognostic Assay Predicts Chemotherapy Benefit in Gastric Cancer: A Multicenter Study. EBioMedicine (2020) 61:103023. 10.1016/j.ebiom.2020.103023 33069062 PMC7569189

[20] Chen XY Liang R Yi YC Fan HN Chen M Zhang J. The m(6)A Reader YTHDF1 Facilitates the Tumorigenesis and Metastasis of Gastric Cancer *via* USP14 Translation in an m(6)A-Dependent Manner. Front Cell Dev Biol (2021) 9:647702. 10.3389/fcell.2021.647702 33791305 PMC8006284

[21] Zhang J Guo S Piao HY Wang Y Wu Y Meng XY. ALKBH5 Promotes Invasion and Metastasis of Gastric Cancer by Decreasing Methylation of the lncRNA NEAT1. J Physiol Biochem (2019) 75:379–89. 10.1007/s13105-019-00690-8 PMC6728298 31290116

[22] Xu D Shao W Jiang Y Wang X Liu Y Liu X. FTO Expression Is Associated With the Occurrence of Gastric Cancer and Prognosis. Oncol Rep (2017) 38:2285–92. 10.3892/or.2017.5904 28849183

[23] Lin S Liu J Jiang W Wang P Sun C Wang X. METTL3 Promotes the Proliferation and Mobility of Gastric Cancer Cells. Open Med (Warsaw Poland) (2019) 14:25–31. 10.1515/med-2019-0005 PMC6419388 30886897

[24] Zhang C Zhang M Ge S Huang W Lin X Gao J. Reduced m6A Modification Predicts Malignant Phenotypes and Augmented Wnt/PI3K-Akt Signaling in Gastric Cancer. Cancer Med (2019) 8:4766–81. 10.1002/cam4.2360 PMC6712480 31243897

[25] Wang Y Cong R Liu S Zhu B Wang X Xing Q. Decreased Expression of METTL14 Predicts Poor Prognosis and Construction of a Prognostic Signature for Clear Cell Renal Cell Carcinoma. Cancer Cell Int (2021) 21:46. 10.1186/s12935-020-01738-2 33430867 PMC7802286

[26] Shi Y Zhuang Y Zhang J Chen M Wu S. METTL14 Inhibits Hepatocellular Carcinoma Metastasis Through Regulating EGFR/PI3K/AKT Signaling Pathway in an m6A-Dependent Manner. Cancer Manage Res (2020) 12:13173–84. 10.2147/CMAR.S286275 PMC7767748 33380825

[27] Dong XF Wang Y Huang BF Hu GN Shao JK Wang Q. Downregulated METTL14 Expression Correlates With Breast Cancer Tumor Grade and Molecular Classification. Biomed Res Int (2020) 2020:8823270. 10.1155/2020/8823270 33134390 PMC7593718

[28] Wang M Liu J Zhao Y He R Xu X Guo X. Upregulation of METTL14 Mediates the Elevation of PERP mRNA N(6) Adenosine Methylation Promoting the Growth and Metastasis of Pancreatic Cancer. Mol Cancer (2020) 19:130. 10.1186/s12943-020-01249-8 32843065 PMC7446161

[29] Tian J Zhu Y Rao M Cai Y Lu Z Zou D. N(6)-Methyladenosine mRNA Methylation of PIK3CB Regulates AKT Signalling to Promote PTEN-Deficient Pancreatic Cancer Progression. Gut (2020) 69:2180–92. 10.1136/gutjnl-2019-320179 32312789

[30] Cao J Mu Q Huang H. The Roles of Insulin-Like Growth Factor 2 mRNA-Binding Protein 2 in Cancer and Cancer Stem Cells. Stem Cells Int (2018) 2018:4217259. 10.1155/2018/4217259 29736175 PMC5874980

[31] Yang Z Wang T Wu D Min Z Tan J Yu B. RNA N6-Methyladenosine Reader IGF2BP3 Regulates Cell Cycle and Angiogenesis in Colon Cancer. J Exp Clin Cancer Research: CR (2020) 39:203. 10.1186/s13046-020-01714-8 32993738 PMC7523351

