# Supplementary material for: The m6A Methyltransferase METTL14-Mediated N6-Methyladenosine Modification of PTEN mRNA Inhibits Tumor Growth and Metastasis in Stomach Adenocarcinoma
Source: Front Oncol. 2021 Aug 12;11:699749. doi: 10.3389/fonc.2021.699749 (PMC8406853; doi:10.3389/fonc.2021.699749)
Supplement: Supplementary file 1 [file Presentation_1.pdf]

Supplement table 1. the Primer sequences for RT-PCR

GENE	Forward Primer	Reverse Primer
METTL14	5'-AGTGCCGACAGCATTGGTG-3'	5'-GGAGCAGAGGTATCATAGGAAGC-3'
PTEN	5'-TGGATTCGACTTAGACTTGACCT-3'	5'-GGTGGGTTATGGTCTTCAAAAGG-3'
GAPDH	5'-TGTGGGCATCAATGGATTTGG-3'	5'-ACACCATGTATTCCGGGTCAAT-3'

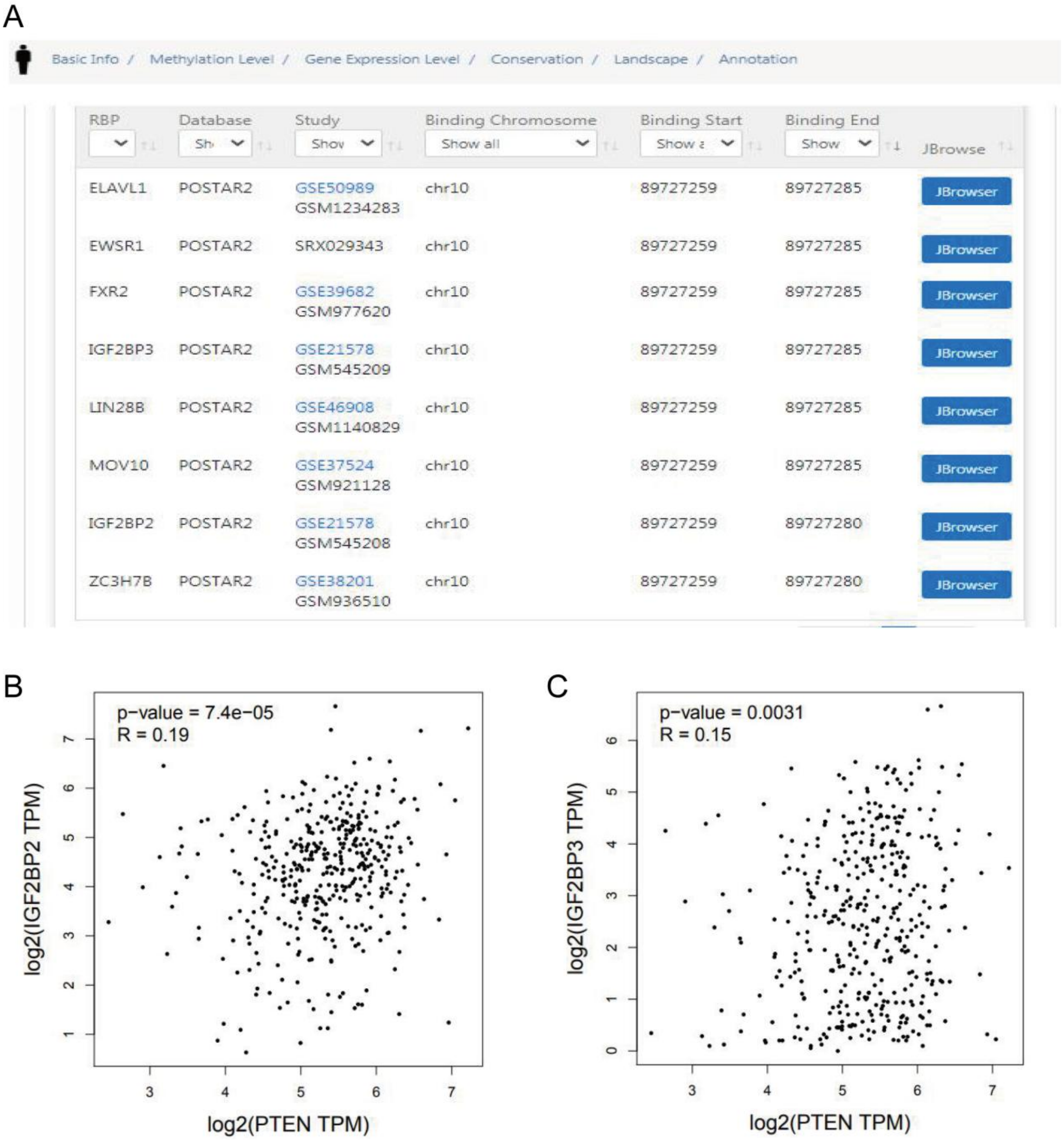

A

Basic Info / Methylation Level / Gene Expression Level / Conservation / Landscape / Annotation

RBP	Database	Study	Binding Chromosome	Binding Start	Binding End	JBrowse
ELAVL1	POSTAR2	GSE50989 GSM1234283	chr10	89727259	89727285	JBrowser
EWSR1	POSTAR2	SRX029343	chr10	89727259	89727285	JBrowser
FXR2	POSTAR2	GSE39682 GSM977620	chr10	89727259	89727285	JBrowser
IGF2BP3	POSTAR2	GSE21578 GSM545209	chr10	89727259	89727285	JBrowser
LIN28B	POSTAR2	GSE46908 GSM1140829	chr10	89727259	89727285	JBrowser
MOV10	POSTAR2	GSE37524 GSM921128	chr10	89727259	89727285	JBrowser
IGF2BP2	POSTAR2	GSE21578 GSM545208	chr10	89727259	89727280	JBrowser
ZC3H7B	POSTAR2	GSE38201 GSM936510	chr10	89727259	89727280	JBrowser

Supplement Figure 1. The potential reader protein of PTEN in STAD. A. M6A-Atlas tool were used to predict PTEN mRNA binding proteins. (http://180.208.58.66/m6A-Atlas/site_info.php?site_ID=human_m6A_20171&id=20171&meRIP=Yes#Annotation). B. Correlation analysis between IGF2BP2 and PTEN mRNA expression in STAD via GEPIA. C. Correlation analysis between IGF2BP3 and PTEN mRNA expression in STAD via GEPIA.
